# URGENT LIVER RETRANSPLANTATION DUE TO TRANSMISSION OF INTRAHEPATIC CHOLANGIOCARCINOMA BY DONOR: THE FIRST REPORT IN THE LITERATURE

**DOI:** 10.1590/0102-672020230022e1740

**Published:** 2023-07-07

**Authors:** Eduardo de Souza Martins Fernandes, Thays Ribeiro Rodrigues de Almeida, Raphael Rodrigues Correa, Eduardo Pinho Braga, Camila Liberato Girão, Leandro Savattone Pimentel, Ronaldo de Oliveira Andrade, Felipe Pedreira Tavares de Mello, Angela Cristina Gouvea Carvalho, Mariana Coelho Peres, Camila Tobias Queiroz, Samanta Teixeira Basto

**Affiliations:** 1 São Lucas Copacabana Hospital, Liver Transplant – Rio de Janeiro (RJ), Brazil; 2 Universidade Federal do Rio de Janeiro, Hospital Universitário Clementino Fraga Filho, Department of Transplant Surgery – Rio de Janeiro (RJ), Brazil; 3 Adventista Silvestre Hospital, Liver transplant – Rio de Janeiro (RJ), Brazil; 4 DASA Hospital São Lucas Copacabana, Department of Pathology – Rio de Janeiro (RJ), Brazil.

**Keywords:** Liver transplant, Cholangitis, sclerosing, Cholangiocarcinoma, Transplante de fígado, Colangite esclerosante, Colangiocarcinoma

## Abstract

**BACKGROUND:**

Liver transplantation represents the best therapeutic modality in end-stage chronic liver disease, severe acute hepatitis, and selected cases of liver tumors.

**AIMS:**

To describe a double retransplant in a male patient diagnosed with Crohn's disease and complicated with primary sclerosing cholangitis, severe portal hypertension, and cholangiocarcinoma diagnosed in the transplanted liver.

**METHODS:**

A 48-year-old male patient diagnosed with Crohn's disease 25 years ago, complicated with primary sclerosing cholangitis and severe portal hypertension. He underwent a liver transplantation in 2018 due to secondary biliary cirrhosis. In 2021, a primary sclerosing cholangitis recurrence was diagnosed and a liver retransplantation was indicated. Recipient's hepatectomy was very difficult by reason of complex portal vein thrombosis requiring extensive thromboendovenectomy. Intraoperative ultrasound with liver doppler evaluation was performed. Two suspicious nodules were incidentally diagnosed in the donor's liver and immediately removed for anatomopathological evaluation.

**RESULTS:**

After pathological confirmation of carcinoma, probable cholangiocarcinoma, at frozen section, the patient was re-listed as national priority and a new liver transplantation was performed within 24 hours. The patient was discharged after 2 weeks.

**CONCLUSIONS:**

The screening for neoplasms in donated organs should be part of our strict daily diagnostic arsenal. Moreover, we argue that, for the benefit of an adequate diagnosis and the feasibility of a safer procedure, the adoption of imaging tests routine for the liver donor is essential, allowing a reduction of the costs and some potential risks of liver transplant procedure.

## RESUMO

**RACIONAL:**

O transplante de fígado representa a melhor modalidade terapêutica na doença hepática crônica terminal, hepatite aguda grave e casos selecionados de tumores hepáticos.

**OBJETIVOS:**

Descrever um retransplante duplo em paciente do sexo masculino, diagnosticado com doença de Crohn e complicado com colangite esclerosante primária, hipertensão portal grave e colangiocarcinoma diagnosticado no fígado transplantado.

**MÉTODOS:**

Paciente do sexo masculino, 48 anos, diagnosticado com doença de Crohn há 25 anos e complicado com colangite esclerosante primária e hipertensão portal grave. Foi submetido a um transplante de fígado em 2018 devido a cirrose biliar secundária. Em 2021, foi diagnosticada recidiva de colangite esclerosante primária e indicado retransplante hepático. A hepatectomia do receptor foi de alta complexidade devido à trombose complexa da veia porta, exigindo extensa tromboendovenectomia. Foi realizada ultrassonografia intraoperatória com doppler hepático. Dois nódulos suspeitos foram diagnosticados incidentalmente no fígado do doador e imediatamente removidos para avaliação anatomopatológica.

**RESULTADOS:**

Após confirmação patológica de carcinoma, provável colangiocarcinoma, pela congelação, o paciente foi relistado como prioridade nacional, e novo transplante hepático foi realizado em 24 horas. O paciente teve alta após 2 semanas.

**CONCLUSÕES:**

O rastreamento de neoplasias em órgãos doados deve fazer parte de nosso estrito arsenal diagnóstico diário. Além disso, defendemos que, em benefício de um diagnóstico correto e da viabilidade de um procedimento mais seguro, a adoção de uma rotina de exames de imagem é essencial em doadores hepáticos, permitindo a redução dos custos e alguns riscos potenciais do procedimento de transplante hepático.

## INTRODUCTION

Liver transplantation (LT) represents the best therapeutic modality in end-stage chronic liver disease, severe acute hepatitis, and selected cases of liver tumors^8^. The transmission of infectious pathogens as well as malignant cells are potent serious complications in the LT. Ultimately, they can negatively impact the patient's evolution, prolong the hospitalization time, and dramatically increase cost^3,7^.

The transmission of malignant cells throughout the organ donation process of a deceased donor has received overwhelming interest worldwide due to its catastrophic consequences. A detailed medical evaluation, a comprehensive clinical history, family interview as well as imaging tests of the potential donor are important tools to recognize and avoid the inadvertent transmission of malignancies in organ transplants^1,3,7^.

### Case report and technique

A 48-year-old male patient was diagnosed with Crohn's disease, 25 years ago. In 2006, the patient underwent a left lateral segmentectomy, owing to intrahepatic lithiasis, without any posterior recurrence. Twelve years later, in 2018, he was referred to the liver transplant clinic due to primary sclerosing cholangitis (PSC) and secondary biliary cirrhosis with severe portal hypertension. During outpatient follow-up, he needed multiple hospitalizations secondary to inflammatory bowel disease exacerbation and acute lower gastrointestinal bleeding. In October 2018, as a consequence of PSC progression, the patient was listed to receive a liver transplant and, 1 month later, underwent a LT without any complications.

Two years after the operation he developed graft cirrhosis, caused by PSC recurrence after liver transplantation. He had several episodes of bacterial cholangitis and was submitted to new liver transplant in 2021. The patient was listed for the second time with featured Model for End-Stage Liver Disease (MELD) score of 35. The second donor was a 69-year-old diabetic female with a BMI of 29.3 kg/m^2^, and who had encephalic death due to a hemorrhagic stroke.

**Figure 1:**
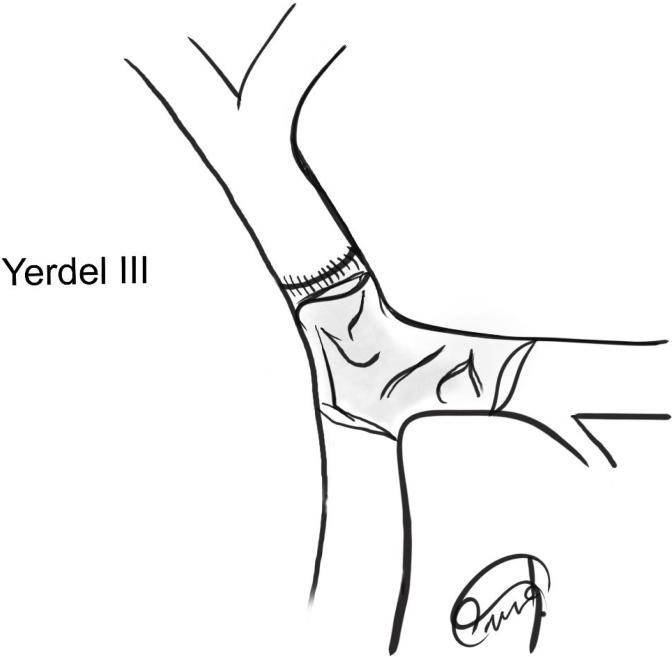
Portal vein thrombosis (Yerdel III).

**Figures 2 and 3:**
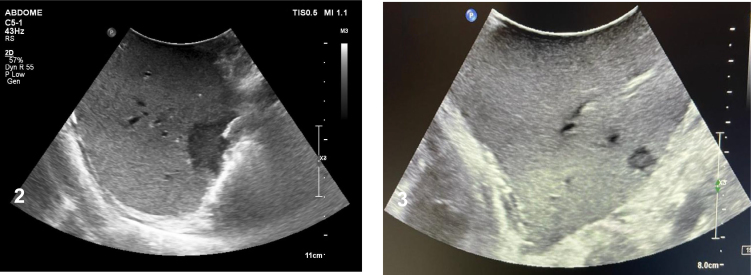
Intraoperative ultrasound lesions on segments VII and segment II.

During the second surgical procedure, a more difficult intraoperative scenario was found caused by a complex portal vein thrombosis (Yerdel III)^9^ (Figure 1), sarcopenia, and previous surgery. After liver reperfusion, an ultrasound with liver doppler detected a hypoechogenic lesion of approximately 2 cm of diameter with a solid aspect and irregular edges located in segment VII (Figure 2) and also, a hypoechogenic and irregular diminutive mass, with approximately 1 cm of diameter in segment II (Figure 3).

**Figures 4 and 5:**
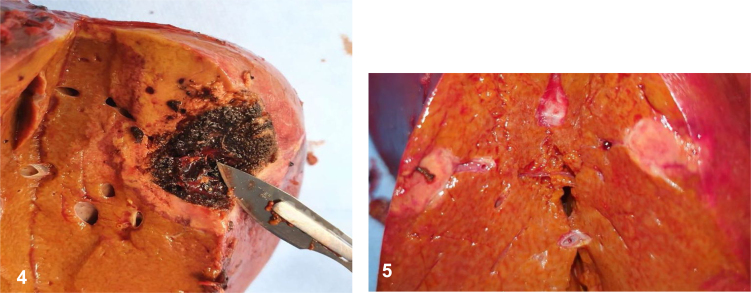
Macroscopy of lesions on segments VII and segment II.

**Figure 6:**
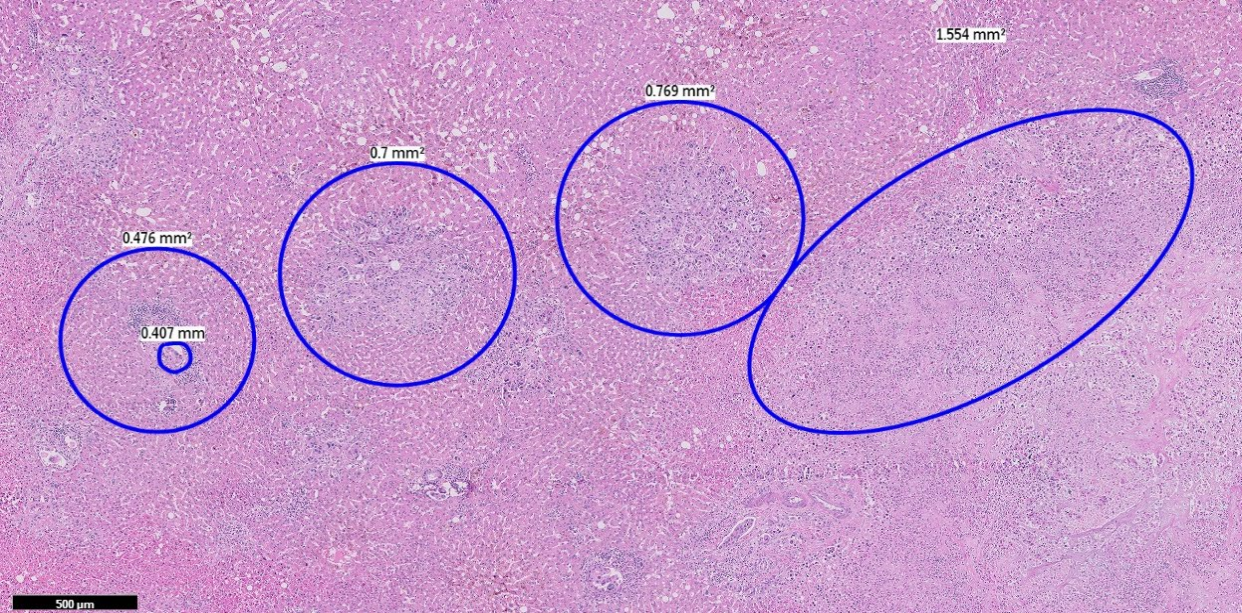
Microscopic section (Hematoxilin & Eosin stain): multiple nodules of intrahepatic cholangiocarcinoma of small duct type.

**Figure 7:**
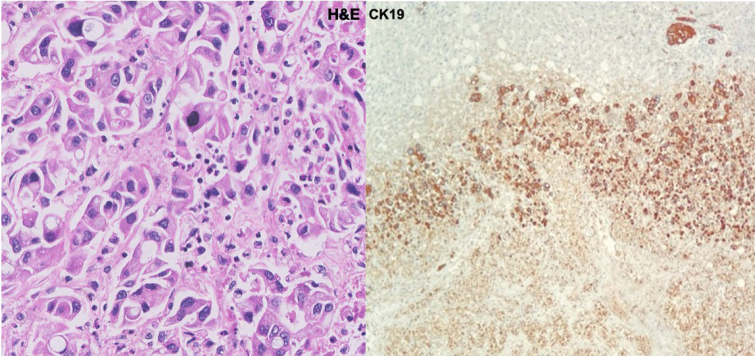
Histological aspect of cholangiocarcinoma – Hematoxylin and Eosin stain (left) and immunohistochemistry positive for CK19 (right).

The enucleation of both lesions was performed (Figures 4 and 5). Frozen section of the largest lesion showed an adenocarcinoma, consistent with cholangiocarcinoma. Gross inspection of the native liver revealed multiple nodules of various sizes, the largest one 0.4 cm. Histologic sections exhibited moderately differentiated intrahepatic cholangiocarcinoma of small duct type (World Health Organization - WHO 2019), with massive lymphovascular invasion (Figure 6). Immunohistochemical markers were helpful to establish this diagnosis, cytokeratin (CK) 7 and CK19 were positive, and although not specific, are usually variably expressed by the biliary tumors (Figure 7). After pathological confirmation, he was re-listed for the third time as a national priority and a new LT was performed within 24 hours. The patient was discharged after two weeks. He has been followed regularly with computed tomography (CT) scan and tumoral markers without signs of tumor recurrence. The patient signed the consent form for this record.

**Figure 8:**
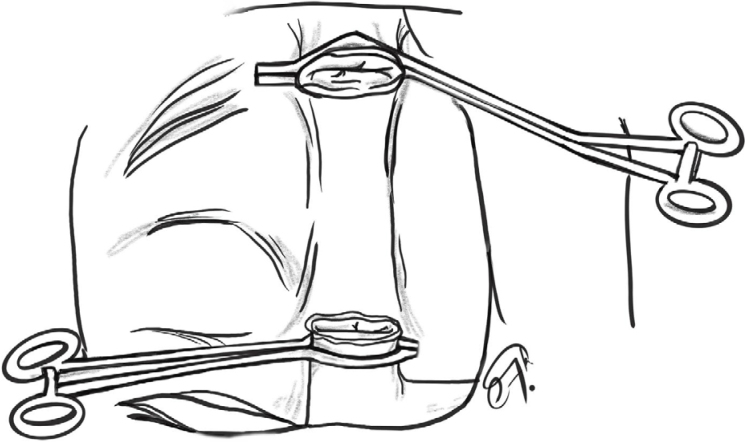
Crossclamp technique – classic bi-caval reconstruction during liver transplantation.

All three liver transplants were performed under the same technique by our team. Total hepatectomy under “classic” technique with vena cava replacement and bicaval anastomosis was performed (Figure 8). During the first transplant, a careful hilar dissection was done with common bile duct excision due to PSC, followed by a hepatic artery ligation above gastroduodenal artery (GDA) and wide exposure of portal vein trunk. Upper and bottom vena cava reconstruction was performed with a running 4.0 Prolene suture, and portal vein anastomosis with running 6.0 Prolene, followed by classical reperfusion. Arterial anastomosis was done with end-to-end anastomosis with a running 7.0 Prolene suture, using a “carrel patch” at recipient hepatic artery and GDA confluence. After meticulous hemostasis, biliary reconstruction was performed using Roux-en-Y hepaticojejunostomy with a running 7.0 Prolene.

**Figure 9:**
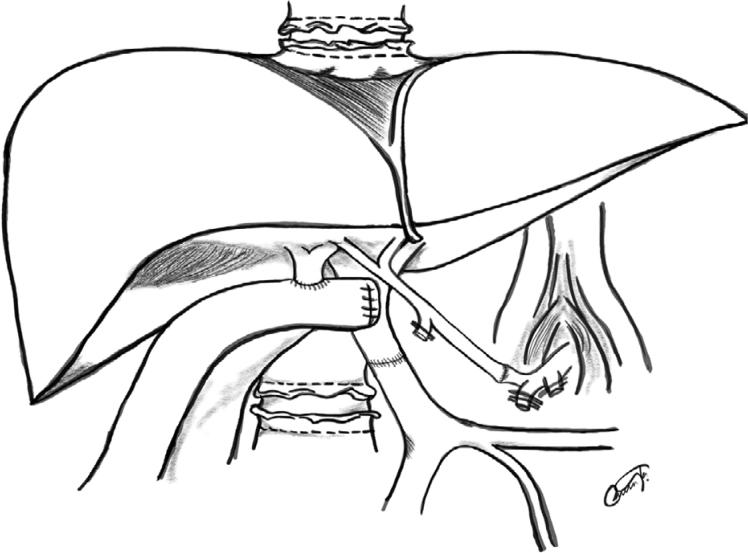
Final aspect after ligation of left gastric artery and splenic artery, celiac artery was dissected in order to guarantee a healthy arterial conduit to provide a perfect arterial flow.

The retransplantation procedure was technically demanding due to severe adhesions, portal hypertension, coagulopathy, and a frozen liver hilum. A massive hilar clamping was necessary to avoid severe bleeding. Extensive thromboendovenectomy was required to achieve adequate portal flow. After the ligation of left gastric artery and splenic artery, celiac artery was dissected in order to guarantee a healthy arterial conduit to provide a perfect arterial flow (Figure 9).

The third retransplantation was uneventful, using the same technique cited above, 48 hours after the second retransplantation with no adhesions and thrombosis.

## DISCUSSION

The transmission of neoplastic cells during the solid organ transplantation process occurs rarely, but when it happens, has major consequences. According to data provided by the Organ Procurement and Transplantation Network/United Network for Organ Share (UNOS), there are a total of 21 cases of malignancy among more than 100,000 cases of solid organ donation^8^. The reported cases showed a higher incidence of lymphomas, melanomas, and neuroendocrine tumors^3,7^.

According to recent reviews, the liver is the most common transplant type associated with transmission of tumor. The most frequently reported malignancies were melanoma (13.6%), choriocarcinoma (9.5%), central nervous system tumors (9.5%), genitourinary tumors (9.5%), hematological malignancies (9.5%), and neuroendocrine tumors (9.5%). In addition, the mortality rate of up to 75% was observed and the mean time to diagnosis was eight months^4,6^. To the best of our knowledge, this is the first report in the current literature on donor transmission of intrahepatic cholangiocarcinoma (CCA).

Cholangiocarcinoma is the second most common primary neoplasm of the liver, after hepatocellular carcinoma. This tumor is more frequent in men aged between 50 to 70 years and the most common clinical presentation is jaundice^5^. Intrahepatic CCA is a type of tumor developed from bile duct cells and is diagnosed through the evaluation of the bile ducts by imaging, histopathological examinations, and hepatic blood screening^5,10^.

Despite the efforts to ensure a healthy organ for transplantation, the transmission of neoplasms from the donor may occur^3,7^. To reduce the risks, some strategies are proposed. Many authors recommend an inspection of the thoracic and abdominal cavities in search of any suspicious nodule or lesion. Besides that, imaging tests for screening patients with a suspected history or within favorable epidemiology should be used as a diagnostic tool. Among all tools available, CT scan have the highest sensitivity and specificity to assist and diagnose possible neoplastic lesions^1^. However, full images of the donated liver are not routinely performed, nor are recommended as part of evaluation process of a cerebral death potential donor.

On top of that, liver transplantation is a highly complex operation that requires both a well-trained specialized team and immeasurable financial investment. The imbalance between offer and need leads to the use of extended criteria donors. The aging of the population coupled with the inefficacy of the public health service are the main contributing factors that lead to many donors who have potential neoplasm lesions being undetected during the donation protocol.

In Brazil, organ donation increased considerably in the last decade^2^. Some remote areas and hospitals with less medical support can potentially under evaluate donors in the full protocol screening for malignant disease. A detailed history might minimize the risks, but does not nullify them.

During donor evaluation, all potential donors undergo laboratory tests for infectious diseases, but there is an undeniable lack of high-standard screening tests and exams for cancer, which could bring severe consequences to the recipient.

## CONCLUSIONS

With our experience in this reported case, we believe that the implementation of imaging tests such as CT scan is a fundamental tool in the evaluation of organ donors. The screening for neoplasms should be part of our strict daily diagnostic arsenal. For all these reasons aforementioned, we strongly believe that it is critical that the current protocol for potential solid organ donors should be reassessed. Moreover, we argue that, for the benefit of a correct diagnosis and the feasibility of a safer procedure, the adoption of imaging tests routine is essential, allowing a reduction of the costs and some potential risks of liver transplant procedure.
